# 2-Eth­oxy-4-[2-(3-nitro­phen­yl)­hydrazono­meth­yl]phenol

**DOI:** 10.1107/S1600536809037908

**Published:** 2009-09-26

**Authors:** Jun-Qiang Chen, Ling Jiang, Shu-Mian Li, Yu-Zhen Chen

**Affiliations:** a Energy Research Institute Co. Ltd, Henan Academy of Sciences, Zhengzhou 450000, People’s Republic of China; b Key Laboratory of Surface and Interface Science of Henan, School of Materials and Chemical Engineering, Zhengzhou University of Light Industry, Zhengzhou 450002, People’s Republic of China

## Abstract

The title Schiff base compound, C_15_H_15_N_3_O_4_, was prepared from a condensation reaction of 3-eth­oxy-4-hydroxy­benz­aldehyde and 3-nitro­phenyl­hydrazine. The mol­ecule is nearly planar; the dihedral angle between the hydroxy­benzene ring and the nitro­benzene ring is 6.57 (7)°. O—H⋯O, O—H⋯N and C—H⋯O hydrogen bonding helps to stabilize the crystal structure.

## Related literature

For applications of Schiff base compounds, see: Kahwa *et al.* (1986 ▶); Santos *et al.* (2001 ▶).
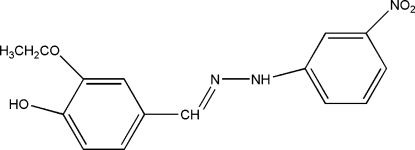


## Experimental

### 

#### Crystal data

C_15_H_15_N_3_O_4_
*M* *_r_* = 301.30
Monoclinic, 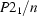

*a* = 12.4160 (6) Å
*b* = 7.7429 (4) Å
*c* = 16.2249 (9) Å
β = 110.497 (6)°
*V* = 1461.04 (13) Å^3^
*Z* = 4
Mo *K*α radiation
μ = 0.10 mm^−1^
*T* = 296 K
0.20 × 0.16 × 0.13 mm

#### Data collection

Bruker SMART CCD area-detector diffractometer
Absorption correction: multi-scan (*SADABS*; Bruker, 1998 ▶) *T*_min_ = 0.979, *T*_max_ = 0.982
5606 measured reflections
2835 independent reflections
1558 reflections with *I* > 2σ(*I*)
*R*_int_ = 0.022

#### Refinement

*R*[*F* ^2^ > 2σ(*F* ^2^)] = 0.035
*wR*(*F* ^2^) = 0.077
*S* = 0.80
2835 reflections
203 parameters
H atoms treated by a mixture of independent and constrained refinement
Δρ_max_ = 0.13 e Å^−3^
Δρ_min_ = −0.16 e Å^−3^

### 

Data collection: *SMART* (Bruker, 1998 ▶); cell refinement: *SAINT* (Bruker, 1998 ▶); data reduction: *SAINT*; program(s) used to solve structure: *SHELXTL* (Sheldrick, 2008 ▶); program(s) used to refine structure: *SHELXTL*; molecular graphics: *SHELXTL*; software used to prepare material for publication: *SHELXTL*.

## Supplementary Material

Crystal structure: contains datablocks global, I. DOI: 10.1107/S1600536809037908/xu2611sup1.cif

Structure factors: contains datablocks I. DOI: 10.1107/S1600536809037908/xu2611Isup2.hkl

Additional supplementary materials:  crystallographic information; 3D view; checkCIF report

## Figures and Tables

**Table 1:** Hydrogen-bond geometry (Å, °)

*D*—H⋯*A*	*D*—H	H⋯*A*	*D*⋯*A*	*D*—H⋯*A*
O2—H2*A*⋯O1	0.89 (2)	2.14 (2)	2.6582 (16)	116.7 (18)
O2—H2*A*⋯N1^i^	0.89 (2)	2.32 (2)	3.0345 (19)	137.0 (15)
C11—H11*A*⋯O2^ii^	0.93	2.56	3.340 (2)	141

Symmetry codes: (i) 
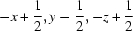
; (ii) 
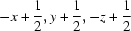
.

**Fig. 1.**
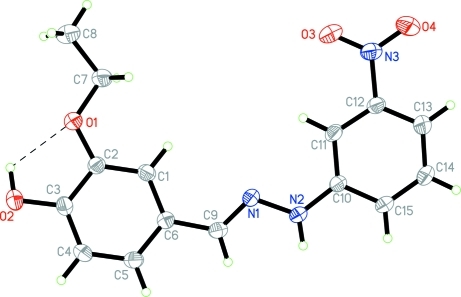
The molecular structure of the title compound with displacement ellipsoids drawn at the 30% probability level, showing intramolecular hydrogen bonds as dashed line.

**Fig. 2.**
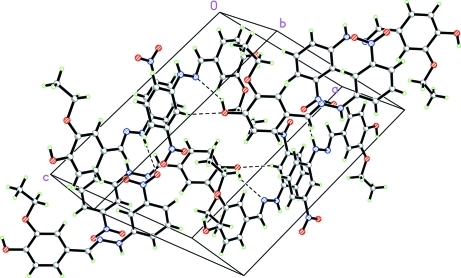
The unit cell packing diagram showing intermolecular hydrogen bonding as dashed lines.

## supplementary crystallographic information

### Comment

The chemistry of Schiff base has attracted a great deal of interest in recent years. These compounds play an important role in the development of various proteins and enzymes (Kahwa *et al.*, 1986; Santos *et al.*, 2001). As part of our study of the coordination chemistry of Schiff bases, we synthesized the title compound and determined its crystal structure.

The molecular structure of (I) is shown in Fig. 1. The hydroxybenzene ring and the nitrobenzene ring is roughly co-planar, making a dihedral angle of 6.57 (7)°. Intramolecular O—H···O hydrogen bond and intermolecular O—H···N and C—H···O hydrogen bonds are observed (Table 1), they help to stabilize the crystal structure (Fig. 2).

### Experimental

2-Nitrophenylhydrazine (1 mmol, 0.153 g) was dissolved in anhydrous ethanol (15 ml). The solution was stirred for several min at 351 K, 3-ethoxy-4-hydroxybenzaldehyde (1 mmol, 0.166 g) in ethanol (8 mm l) was added dropwise and the mixture was stirred at refluxing temperature for 2 h. The solid product was isolated and recrystallized from methanol, red single crystals were obtained after 3 d.

### Refinement

Hydroxy H atom was located in a difference Fourier map and refined isotropically. The other H atoms were positioned geometrically and refined as riding with C—H = 0.93 (aromatic), 0.97 (methylene), 0.96 Å (methyl) and N—H = 0.86 Å, with *U*_iso_(H) = 1.5*U*_eq_(C) for methyl H and *U*_iso_(H) = 1.2*U*_eq_(C,N).

### Crystal data

**Table:**

C_15_H_15_N_3_O_4_	*F*(000) = 632
*M**_r_* = 301.30	*D*_x_ = 1.370 Mg m^−^^3^
Monoclinic, *P*2_1_/*n*	Mo *K*α radiation, λ = 0.71073 Å
Hall symbol: -P 2yn	Cell parameters from 1850 reflections
*a* = 12.4160 (6) Å	θ = 3.2–28.2°
*b* = 7.7429 (4) Å	µ = 0.10 mm^−^^1^
*c* = 16.2249 (9) Å	*T* = 296 K
β = 110.497 (6)°	Block, red
*V* = 1461.04 (13) Å^3^	0.20 × 0.16 × 0.13 mm
*Z* = 4	

### Data collection

**Table:**

Bruker SMART CCD area-detector diffractometer	2835 independent reflections
Radiation source: fine-focus sealed tube	1558 reflections with *I* > 2σ(*I*)
graphite	*R*_int_ = 0.022
ω scans	θ_max_ = 26.0°, θ_min_ = 3.2°
Absorption correction: multi-scan (*SADABS*; Bruker, 1998)	*h* = −15→15
*T*_min_ = 0.979, *T*_max_ = 0.982	*k* = −9→8
5606 measured reflections	*l* = −20→10

### Refinement

**Table:**

Refinement on *F*^2^	Primary atom site location: structure-invariant direct methods
Least-squares matrix: full	Secondary atom site location: difference Fourier map
*R*[*F*^2^ > 2σ(*F*^2^)] = 0.035	Hydrogen site location: inferred from neighbouring sites
*wR*(*F*^2^) = 0.077	H atoms treated by a mixture of independent and constrained refinement
*S* = 0.80	*w* = 1/[σ^2^(*F*_o_^2^) + (0.0412*P*)^2^] where *P* = (*F*_o_^2^ + 2*F*_c_^2^)/3
2835 reflections	(Δ/σ)_max_ < 0.001
203 parameters	Δρ_max_ = 0.13 e Å^−^^3^
0 restraints	Δρ_min_ = −0.16 e Å^−^^3^

### Special details

**Table:**

Geometry. All e.s.d.'s (except the e.s.d. in the dihedral angle between two l.s. planes) are estimated using the full covariance matrix. The cell e.s.d.'s are taken into account individually in the estimation of e.s.d.'s in distances, angles and torsion angles; correlations between e.s.d.'s in cell parameters are only used when they are defined by crystal symmetry. An approximate (isotropic) treatment of cell e.s.d.'s is used for estimating e.s.d.'s involving l.s. planes.
Refinement. Refinement of *F*^2^ against ALL reflections. The weighted *R*-factor *wR* and goodness of fit *S* are based on *F*^2^, conventional *R*-factors *R* are based on *F*, with *F* set to zero for negative *F*^2^. The threshold expression of *F*^2^ > σ(*F*^2^) is used only for calculating *R*-factors(gt) *etc*. and is not relevant to the choice of reflections for refinement. *R*-factors based on *F*^2^ are statistically about twice as large as those based on *F*, and *R*- factors based on ALL data will be even larger.

### Fractional atomic coordinates and isotropic or equivalent isotropic displacement parameters (Å^2^)

**Table:**

	*x*	*y*	*z*	*U*_iso_*/*U*_eq_	
O1	0.13682 (9)	0.87732 (14)	0.23604 (7)	0.0531 (3)	
O2	0.22167 (11)	0.78440 (15)	0.40430 (8)	0.0574 (3)	
N1	0.50471 (10)	1.09280 (16)	0.17334 (8)	0.0449 (3)	
C6	0.44686 (13)	0.95951 (18)	0.28540 (10)	0.0400 (4)	
C10	0.58320 (12)	1.20825 (19)	0.06891 (10)	0.0408 (4)	
C11	0.47700 (12)	1.22659 (19)	0.00284 (10)	0.0424 (4)	
H11A	0.4099	1.1937	0.0117	0.051*	
N3	0.36075 (12)	1.31235 (19)	−0.14578 (10)	0.0586 (4)	
C2	0.25328 (13)	0.89131 (18)	0.27554 (10)	0.0403 (4)	
C4	0.40865 (14)	0.86218 (19)	0.41319 (10)	0.0498 (4)	
H4A	0.4353	0.8353	0.4728	0.060*	
C5	0.48479 (13)	0.91857 (19)	0.37376 (10)	0.0467 (4)	
H5A	0.5625	0.9290	0.4072	0.056*	
N2	0.59659 (11)	1.14119 (17)	0.15028 (9)	0.0566 (4)	
H2B	0.6649	1.1291	0.1881	0.068*	
C9	0.53084 (13)	1.02074 (18)	0.24853 (10)	0.0449 (4)	
H9A	0.6083	1.0066	0.2817	0.054*	
C1	0.32963 (13)	0.94411 (18)	0.23613 (10)	0.0415 (4)	
H1B	0.3030	0.9697	0.1763	0.050*	
C12	0.47362 (12)	1.29476 (19)	−0.07618 (10)	0.0418 (4)	
C3	0.29426 (14)	0.84598 (18)	0.36459 (10)	0.0424 (4)	
C15	0.68158 (13)	1.2571 (2)	0.05225 (11)	0.0525 (4)	
H15A	0.7534	1.2441	0.0959	0.063*	
O3	0.27636 (11)	1.2634 (2)	−0.13242 (10)	0.1076 (6)	
C13	0.56883 (14)	1.3452 (2)	−0.09402 (11)	0.0545 (5)	
H13A	0.5630	1.3915	−0.1483	0.065*	
O4	0.35438 (11)	1.3746 (2)	−0.21577 (9)	0.0961 (5)	
C7	0.08594 (14)	0.9091 (2)	0.14407 (11)	0.0570 (5)	
H7A	0.1026	0.8145	0.1112	0.068*	
H7B	0.1167	1.0146	0.1288	0.068*	
C14	0.67357 (14)	1.3242 (2)	−0.02778 (12)	0.0627 (5)	
H14A	0.7403	1.3562	−0.0376	0.075*	
C8	−0.04114 (14)	0.9256 (2)	0.12228 (12)	0.0676 (5)	
H8A	−0.0776	0.9469	0.0604	0.101*	
H8B	−0.0567	1.0200	0.1549	0.101*	
H8C	−0.0707	0.8206	0.1376	0.101*	
H2A	0.1526 (18)	0.777 (2)	0.3624 (14)	0.100 (8)*	

### Atomic displacement parameters (Å^2^)

**Table:**

	*U*^11^	*U*^22^	*U*^33^	*U*^12^	*U*^13^	*U*^23^
O1	0.0394 (6)	0.0719 (8)	0.0458 (7)	−0.0045 (5)	0.0122 (5)	0.0102 (6)
O2	0.0559 (8)	0.0773 (8)	0.0446 (8)	−0.0088 (7)	0.0246 (7)	0.0017 (6)
N1	0.0349 (7)	0.0587 (8)	0.0418 (8)	−0.0058 (6)	0.0142 (6)	0.0001 (7)
C6	0.0386 (9)	0.0409 (9)	0.0397 (10)	−0.0013 (7)	0.0127 (8)	−0.0005 (7)
C10	0.0327 (9)	0.0507 (9)	0.0379 (10)	−0.0041 (7)	0.0112 (7)	−0.0017 (7)
C11	0.0310 (9)	0.0550 (10)	0.0429 (10)	−0.0045 (7)	0.0152 (8)	−0.0059 (8)
N3	0.0406 (9)	0.0810 (10)	0.0471 (10)	0.0004 (8)	0.0064 (8)	0.0040 (8)
C2	0.0376 (9)	0.0406 (9)	0.0416 (10)	−0.0012 (7)	0.0125 (8)	0.0015 (7)
C4	0.0519 (11)	0.0609 (11)	0.0333 (10)	−0.0019 (9)	0.0107 (8)	0.0043 (8)
C5	0.0395 (9)	0.0525 (10)	0.0420 (10)	−0.0015 (8)	0.0065 (8)	0.0020 (8)
N2	0.0293 (7)	0.0921 (11)	0.0443 (9)	−0.0077 (7)	0.0078 (6)	0.0119 (8)
C9	0.0349 (9)	0.0529 (10)	0.0421 (11)	−0.0007 (8)	0.0074 (8)	−0.0006 (8)
C1	0.0428 (9)	0.0441 (9)	0.0345 (9)	−0.0022 (7)	0.0098 (7)	0.0017 (7)
C12	0.0338 (9)	0.0508 (10)	0.0387 (10)	−0.0019 (7)	0.0101 (7)	−0.0044 (7)
C3	0.0469 (10)	0.0445 (9)	0.0391 (10)	−0.0024 (8)	0.0193 (8)	−0.0002 (7)
C15	0.0301 (9)	0.0729 (12)	0.0501 (11)	−0.0061 (8)	0.0087 (8)	0.0071 (9)
O3	0.0362 (8)	0.1872 (16)	0.0859 (11)	−0.0113 (9)	0.0045 (7)	0.0440 (10)
C13	0.0493 (11)	0.0695 (12)	0.0447 (11)	−0.0089 (9)	0.0165 (9)	0.0063 (8)
O4	0.0645 (9)	0.1613 (15)	0.0501 (9)	−0.0042 (8)	0.0043 (7)	0.0324 (9)
C7	0.0453 (10)	0.0696 (11)	0.0488 (11)	−0.0090 (9)	0.0071 (9)	0.0076 (9)
C14	0.0378 (10)	0.0927 (14)	0.0594 (13)	−0.0133 (9)	0.0192 (9)	0.0098 (10)
C8	0.0471 (11)	0.0812 (12)	0.0652 (13)	0.0020 (10)	0.0081 (9)	0.0044 (10)

### Geometric parameters (Å, °)

**Table:**

O1—C2	1.3654 (16)	C4—C3	1.368 (2)
O1—C7	1.4234 (18)	C4—C5	1.385 (2)
O2—C3	1.3643 (18)	C4—H4A	0.9300
O2—H2A	0.89 (2)	C5—H5A	0.9300
N1—C9	1.2759 (17)	N2—H2B	0.8600
N1—N2	1.3713 (16)	C9—H9A	0.9300
C6—C5	1.380 (2)	C1—H1B	0.9300
C6—C1	1.3989 (19)	C12—C13	1.369 (2)
C6—C9	1.451 (2)	C15—C14	1.369 (2)
C10—N2	1.3735 (19)	C15—H15A	0.9300
C10—C11	1.385 (2)	C13—C14	1.376 (2)
C10—C15	1.393 (2)	C13—H13A	0.9300
C11—C12	1.374 (2)	C7—C8	1.497 (2)
C11—H11A	0.9300	C7—H7A	0.9700
N3—O3	1.2031 (17)	C7—H7B	0.9700
N3—O4	1.2105 (17)	C14—H14A	0.9300
N3—C12	1.4660 (19)	C8—H8A	0.9600
C2—C1	1.3782 (19)	C8—H8B	0.9600
C2—C3	1.398 (2)	C8—H8C	0.9600
			
C2—O1—C7	119.12 (12)	C2—C1—C6	120.48 (14)
C3—O2—H2A	106.4 (14)	C2—C1—H1B	119.8
C9—N1—N2	115.03 (13)	C6—C1—H1B	119.8
C5—C6—C1	118.84 (14)	C13—C12—C11	124.11 (15)
C5—C6—C9	118.04 (14)	C13—C12—N3	118.29 (15)
C1—C6—C9	123.11 (14)	C11—C12—N3	117.60 (14)
N2—C10—C11	123.01 (14)	O2—C3—C4	118.96 (15)
N2—C10—C15	118.04 (14)	O2—C3—C2	120.94 (14)
C11—C10—C15	118.94 (14)	C4—C3—C2	120.09 (14)
C12—C11—C10	118.13 (14)	C14—C15—C10	120.65 (15)
C12—C11—H11A	120.9	C14—C15—H15A	119.7
C10—C11—H11A	120.9	C10—C15—H15A	119.7
O3—N3—O4	121.30 (16)	C12—C13—C14	116.81 (15)
O3—N3—C12	119.33 (15)	C12—C13—H13A	121.6
O4—N3—C12	119.37 (15)	C14—C13—H13A	121.6
O1—C2—C1	126.38 (14)	O1—C7—C8	107.85 (14)
O1—C2—C3	114.05 (13)	O1—C7—H7A	110.1
C1—C2—C3	119.57 (14)	C8—C7—H7A	110.1
C3—C4—C5	120.11 (15)	O1—C7—H7B	110.1
C3—C4—H4A	119.9	C8—C7—H7B	110.1
C5—C4—H4A	119.9	H7A—C7—H7B	108.4
C6—C5—C4	120.84 (15)	C15—C14—C13	121.35 (15)
C6—C5—H5A	119.6	C15—C14—H14A	119.3
C4—C5—H5A	119.6	C13—C14—H14A	119.3
N1—N2—C10	122.22 (13)	C7—C8—H8A	109.5
N1—N2—H2B	118.9	C7—C8—H8B	109.5
C10—N2—H2B	118.9	H8A—C8—H8B	109.5
N1—C9—C6	123.92 (14)	C7—C8—H8C	109.5
N1—C9—H9A	118.0	H8A—C8—H8C	109.5
C6—C9—H9A	118.0	H8B—C8—H8C	109.5

### Hydrogen-bond geometry (Å, °)

**Table:**

*D*—H···*A*	*D*—H	H···*A*	*D*···*A*	*D*—H···*A*
O2—H2A···O1	0.89 (2)	2.14 (2)	2.6582 (16)	116.7 (18)
O2—H2A···N1^i^	0.89 (2)	2.32 (2)	3.0345 (19)	137.0 (15)
C11—H11A···O2^ii^	0.93	2.56	3.340 (2)	141

Symmetry codes: (i) −*x*+1/2, *y*−1/2, −*z*+1/2; (ii) −*x*+1/2, *y*+1/2, −*z*+1/2.

## References

[bb1] Bruker (1998). *SMART*, *SAINT* and *SADABS* Bruker AXS Inc., Madison, Wisconsin, USA.

[bb2] Kahwa, I. A., Selbin, I., Hsieh, T. C. Y. & Laine, R. A. (1986). *Inorg. Chim. Acta*, **118**, 179–185.

[bb3] Santos, M. L. P., Bagatin, I. A., Pereira, E. M. & Ferreira, A. M. D. C. (2001). *J. Chem. Soc. Dalton Trans.* pp. 838–844.

[bb4] Sheldrick, G. M. (2008). *Acta Cryst.* A**64**, 112–122. 10.1107/S0108767307043930 18156677

